# Development of post-disaster psychosocial evaluation and intervention for children: Results of a South Korean delphi panel survey

**DOI:** 10.1371/journal.pone.0195235

**Published:** 2018-03-29

**Authors:** Mi-Sun Lee, Jun-Won Hwang, Cheol-Soon Lee, Ji-Youn Kim, Ju-Hyun Lee, Eunji Kim, Hyoung Yoon Chang, SeungMin Bae, Jang-Ho Park, Soo-Young Bhang

**Affiliations:** 1 Department of Psychiatry, Eulji University Hospital, Seoul, Korea; 2 Department of Psychiatry, Kangwon National University School of Medicine, Chuncheon, Korea; 3 Department of Psychiatry, Gyeongsang National University School of Medicine, Gyeongsang National University Changwon Hospital, Changwon, Korea; 4 Goodmind Psychiatry Clinic, Suwon, Korea; 5 Inarae Psychiatry Clinic, Seoul, Korea; 6 Todak Psychiatry Clinic, Ansan, Korea; 7 Department of Psychiatry, Ajou University School of Medicine, Suwon, Korea; 8 Sunflower Center of Southern Gyeonggi for Women and Children Victims of Violence, Suwon, Korea; 9 Department of Psychiatry, Gachon University Gil Medical Center, Incheon, Korea; 10 Department of Psychiatry, Ulsan University Hospital, University of Ulsan College of Medicine, Ulsan, Korea; 11 Department of Psychiatry, Eulji University School of Medicine, Eulji University Hospital, Seoul, Korea; Johns Hopkins University Bloomberg School of Public Health, UNITED STATES

## Abstract

**Objective:**

This study aimed to administer a Delphi panel survey and provide evidence for the development of a psychological intervention protocol for use after disasters in South Korea.

**Method:**

A three-round Delphi survey was conducted. In all rounds, respondents answered open- or closed-ended questions regarding their views on i) the concept of disaster, ii) evaluation, iii) intervention, and iv) considerations in a disaster. Data from Round 1 were subjected to content analysis. In Round 2, items with content validity ratios (CVRs) greater than 0.49 were included, and in Round 3, items with a CVR≥0.38 were accepted.

**Results:**

The response rates for the Delphi survey were high: 83% (n = 15, Round 1), 80% (n = 16, Round 2), and 86% (n = 24, Round 3). The data collected during this survey showed a need for a support system for children; for preventive strategies, including disaster readiness plans; for the protection of children's safety; and for the development of post-disaster psychosocial care.

**Conclusions:**

The panel experts reached a consensus regarding the steps they considered critical in post-disaster evaluation and intervention. The findings suggest a unified model for advancing the development of the Korean version of an intervention protocol for children and adolescents exposed to traumatic events.

## Introduction

Natural and man-made disasters are common worldwide. Various disasters have occurred in South Korea in the twenty-first century, such as typhoons, floods, and subway fires. Among them is the sinking of the Sewol ferry, which occurred on the morning of April 16, 2014. The ferry capsized while carrying 476 people, mostly secondary school students from Danwon High School. In total, 304 passengers and crew members died in the disaster. The Sewol ferry disaster severely shocked Korean society and resulted in widespread social and political reactions in South Korea.

Traumatic symptoms in children and adolescents are expressed in a variety of forms depending on their developmental stage. Children can develop PTSD (Post-Traumatic Stress Disorder) and other mental health problems following traumatic events.[1] Moreover, a significant minority of children who are particularly vulnerable have ongoing difficulties.[2] Compared with studies of adult samples, studies of youth outcomes after a disaster generally report higher estimates for the prevalence of mental health disorders.[3] Therefore, to help children and adolescents, it is very important to evaluate and intervene in situations of psychological trauma.

In South Korea, before April 16, 2014, there were no efforts to prepare the population for coping with disaster. Systematic psychological intervention guides for disaster situations have never been provided.

We searched through guidelines such as the WHO guidelines[4], the Mental Health Gap Action Programme (mhGAP) Humanitarian Intervention Guide[5] and recommendations by the Inter-Agency Standing Committee (IASC)[6]. However, the use of available practical guidelines for disaster and trauma patients might be limited due to cultural differences in medical situations and clinical environments. Therefore, protocols that can more aptly respond to culturally specific situations and issues in South Korea are required.[7] The country has suffered from a lack of crisis intervention approaches to follow after disasters. For these reasons, confusion arose when the sinking of MV Sewol occurred on April 16, 2014. Therefore, we seek to study and suggest practical directions for establishing guidelines in South Korea.

In this regard, a Delphi study for disaster care is necessary. The Delphi methodology is a widely used group survey technique typically conducted in three consecutive rounds to evaluate consensus among experts in a field. The quality of the panel of experts and their opinions on the given topic is considered a strength of the Delphi technique.[8] The approach has the advantage of obtaining expert opinion with a guarantee of anonymity, thus avoiding potential distortion caused by peer pressure in group situations such as focus group analysis.[9] Above all, this technique is most effective when there is a lack of information or only inadequate information on a particular issue.

In this context, it is particularly important to monitor the psychosocial care guidelines for children after a disaster. However, to our knowledge, no researchers have examined expert opinion via a Delphi study in post-disaster situations in South Korea.

This survey details the design of a Delphi study for addressing appropriate psychosocial care guidelines for children and adolescents after a disaster. The agreed-upon measures could constitute a standardized approach to initial clinical evaluation and intervention to help identify individuals in need after a disaster.[10] A three-round Delphi study was undertaken to elicit a prioritized list of research topics to guide future research efforts and thus obtain meaningful results.[11] Consequently, using the Delphi survey technique, this study aimed to evaluate the usefulness and direction of the development of post-traumatic assessment and intervention based on the opinions of pediatric and disaster- and trauma-related experts.

## Methods

The Delphi study consisted of three consultation rounds from January to May 2016. In each Delphi round, we provided the panel with feedback on the results of the previous consultation, and routine communications with panel experts were conducted by e-mail. The study was approved by Eulji University's Institutional Review Board (IRB No. EMCS 2015-12-004).

### Delphi study

A Delphi study is a structured process that invites experts to complete a series of ‘rounds’ to gather and refine information related to the study question until an expert consensus is reached.[12] A commonly used formal consensus method is the Delphi technique, which involves two or more rounds of postal or online questionnaires.[13]

According to previous studies, two or three rounds are frequently used in the Delphi process.[12] The survey rounds interactively ask experts to prioritize issues or rate them on implementation-related scales, such as scales measuring feasibility or desirability, enabling controlled feedback on the previous round’s group results.[14] This group facilitation technique aims to obtain consensus among the opinions of ‘experts’ through a series of structured questionnaires.[15]

### Delphi panel

A Delphi study is conducted with a group of individuals considered to have expertise (both professional and experience-based) in the field under investigation.[16] The Delphi panel in this study consisted of experts in child and adolescent mental health, professionals providing disaster psychological support, and related practitioners with experience in disasters. Our survey included a range of mental health professionals.[13]

The Delphi technique allows for the selection of experts and does not require a representative sample of the population. We note that the literature on Delphi surveys traditionally recommends a panel of 10 to 15 experts, typical of most qualitative research.[17] However, a panel size ranging from 20 to 50 has been deemed appropriate.[18] Therefore, the present study is informed by recommendations of a sample size from 10 to 50 for qualitative research and Delphi surveys designed to generate hypotheses.[19]

The Delphi panel participants were also required to provide basic demographic information and professional characteristics.[20] Anonymity was assured for all participants during the study; anonymity prevents the influence of the authority, status, personality, or reputation of group members in the process, thereby preventing biased outcomes.[21]

### First-round questionnaire

The Round 1 survey consisted of 20 open-ended questions grouped into four themes (S1 Appendix). Several open-ended questions were included to ensure that the survey accommodated the opinions of professionals from a multidisciplinary team. After confirming participation, panel participants were e-mailed an invitation to activate the Round 1 questionnaire. We conducted the online interview and received informed consent from all participants on the expert panel before interviewing them. The responses had no word limits, and participants were encouraged to give their opinions freely. Reminders were sent if the survey had not been returned. The survey was open for one month.

### Second-round questionnaire

Questions for Rounds 2 were developed based on the participants' responses in the previous round. Converged answers in Round 1 were classified as evaluation and intervention, and freely presented expert opinions were based on detailed questions. The Round 2 survey consisted of 156 closed-ended questions with responses grouped into 27 themes. The experts received the second-round questionnaire by e-mail and were instructed to rate and score the importance of each indicator on a five-point Likert scale (1 = very unimportant, 3 = neutral and 5 = very important). An item was considered important if ≥80% of the respondents awarded it a score of 4 or 5; otherwise, the item was removed. The experts were encouraged to provide comments freely on each indicator and/or to propose indicators that they considered important. Routine communication with panel experts was conducted by e-mail.

### Third-round questionnaire

Round 3 excluded 44 items that did not receive a consensus in Round 2. For 112 items, 80% agreement was reached. In Round 2, the experts freely commented on each indicator that they considered important. Based on these responses, 11 items were modified, and 63 items were added.

Ultimately, 175 items were composed and grouped into 25 themes. In the third round, we asked the panel to rate the importance of each topic on a 5-point Likert scale from 1 (not important) to 5 (very important). The level of consensus was set to 80% of respondents indicating agreement.[9] Individual and anonymous opinions were solicited via e-mail.

### Data analysis

Delphi questionnaires were coded individually. Members of the research team alone had access to the codes to facilitate follow-up. Any published data identified individuals, their institution, or organizations.

In Round 1, all topics suggested by the panel experts were categorized using content analysis. We identified words or expressions in conceptual categories to understand and identify the relationships among themes. We performed categorization by removing irrelevant, overlapping and repeated content; looking for common viewpoints; and identifying responses. To analyze the Round 2 and 3 responses, we calculated content validity ratios (CVRs). The minimum CVR was determined by the number of experts participating in each round.

We used the formula CVR = (n_e_ -*N*/2)/ (*N*/2), where n_e_ represents the number of panel experts rating an item as ‘essential’ (score of 4 or 5) and *N* represents the entire number of panelists.[22] The CVR ranges from +1 to −1. A high positive value indicates that the survey experts agreed that a factor or item was essential.[23]

Therefore, in Round 2, the CVR values of all items were set to 0.49 for the 16 panels. Additionally, in Round 3, the minimum CVR value was set to 0.38 for the 24 panels.

## Results

### Demographics of the panel experts

The demographic characteristics of the experts are described in Table 1.

**Table 1:** Demographic characteristics of the panel experts.

	Round 1 (N = 15)		Round 2 (N = 16)		Round 3 (N = 24)	
	N	%	N	%	N	%
Participant response rate	15/18 (83.00)		16/20 (80.00)		24/28 (86.00)	
Age, mean (SD)	44.07 (6.84)		43.75 (7.14)		43.83 (8.33)	
Gender						
Male	5	(33.33)	5	(31.25)	5	(20.83)
Female	10	(66.67)	11	(68.75)	19	(79.17)
Education level						
Bachelor's degree	2	(13.33)	1	(6.25)	1	(4.16)
Master's degree	1	(6.67)	3	(18.75)	4	(16.67)
Doctoral course	2	(13.33)	3	(18.75)	4	(16.67)
Ph.D.	10	(66.67)	9	(56.25)	15	(62.50)
Profession						
Psychiatrist	15	(100.00)	10	(62.50)	17	(70.84)
Psychologist	0	(0.00)	5	(31.25)	5	(20.83)
Social worker	0	(0.00)	1	(6.25)	2	(8.33)

SD: standard deviation

In Round 1, 18 experts registered to be members of the Delphi panel, and 15 of them (83%) (10 female, 5 males) returned the Round 1 questionnaire. The mean age of the experts was 44.07 years (standard deviation: 6.84 years). Approximately 10 (66.67%) of the respondents had earned a Ph.D.

In Round 2, 20 participants were included, and 16 (80%) responded; the respondents included psychiatrists (10), psychologists (5), and a social worker (1). The mean age of the experts was 43.75 years (standard deviation: 7.14 years). Approximately 11 (68.75%) of the panel experts were women, and 9 (56.25%) had earned a Ph.D. as their highest level of education.

In Round 3, 28 psychiatric professionals registered to be members of the expert panel, and 24 (86%) returned the questionnaires. The mean age of the experts was 43.83 years (standard deviation: 8.33 years); the experts included psychiatrists (17), psychologists (5), and social workers (2). Most of the experts were females (19), and 15 (62.50%) had earned a Ph.D. Round 3 experts showed an adequate level of agreement on the research topics (Table 1).

### Results of first-round Delphi survey

Qualitative content analysis was used in Round 1. The Round 1 results are described in detail in a previously published paper.[24] We found that the following issues have a strong effect on post-disaster interventions: proper timing of the initial interview in the event of a disaster, assessment notification, assessment services for individuals, mandatory enforcement measures, scale screening and treatment intervention elements, symptom degree classification, intervention standardization, program level, care unit environments, and operation plans.

The table in the preliminary research paper that included the Round 1 items and content has been reproduced. We sought permission from previous journals to re-use the table and to add a reference (Table 2).

**Table 2:** Categories and items of the first round of the Delphi study*.

Categories	Items
I. Currently used child-adolescent assessment and treatment protocols in disasters	Treatment programs that have been proven to be effective in previous disasters
	Difficulties when implementing assessment protocols and treatment programs in disasters
	Need to promote previous child-adolescent treatment programs
II. Direction of child-adolescent assessment protocols after disaster	Need for child-adolescent psychological assessment intervention after disaster
	Adequate means of psychological assessment procedures
	Constructing an environment for psychological assessments
	Things to consider when using brief scales
	Essential factors when selecting assessment scales
III. Direction of child-adolescent treatment programs after disasters	Critical factors in child-adolescent treatment intervention after disasters
	Timeframe for treatment program intervention and its evidence
	Timeframe for treatment program termination and its evidence
	Adequate treatment programs for children and adolescents
	Means of operating treatment programs
	Need for standardization of the Korean version of foreign treatment programs
IV. Things to consider in disaster interventions	Level and qualifications of treatment professionals
	Current level of continuing education system construction for child-adolescent disaster professionals
	Ways for disaster professionals to continuously participate in treatment
	Effective ways of promoting treatment programs

*We refer to the table in the previous study.[24]

### Results of second-round Delphi survey

The categories and items on the Delphi panel survey are described in Table 3.

**Table 3:** Categories and items of the second and third rounds of the Delphi study.

Categories	Items	Details	
Concept of child- Adolescent trauma in disasters	1. Concept of trauma in disasters	1) Unique model for other psychopathologies	
		2) Child-adolescent trauma after disaster	
	2. Recovery of trauma in disasters	1) Return to the daily lives of children and adolescents	
		2) Stabilization of developmental tasks (academic function, peer relationships)	
Child-adolescent assessment after disasters	1. Baseline psychological assessments	1) Importance of assessment	
		2) Intake and screening	(1) Critical factors in screening
			(2) Children and adolescents
			(2) Adequate time for screening
			(3) Things to consider for screening
		3) Developmental recording	(1) Things to include in the developmental record of children and adolescents
			(2) Providing assessment service by age
	2. Constructing psychological assessments	1) Constructing an environment for psychological assessments	
		2) Means of operating assessment	
		3) Scales recommended for universal screening	(1) Trauma-related scale
			(2) Depression/anxiety scale
			(3) Overall emotion/behavior scale
			(4) Family-related scale
			(5) Intelligence test
			(6) Neuropsychological test
			(7) Other scales
		4) Things to consider in selecting a scale	(1) Adequate number of scales
			(2) Each number of scale items
			(3) Appropriate age of children and adolescents
		5) Storage and maintenance of scales and analysis report	
	3. Assessment professionals	1) Application plan for disaster assessment professionals	
		2) Professionals	(1) Level of assessment professionals
			(2) Qualification of assessment professionals
		3) Arrangement of child-adolescent disaster assessment professionals	
		4) Education system construction for child-adolescent disaster professionals	
	4. Promoting assessments	1) Participation in assessment	(1) Ways for system to continuously participate in assessment
			(2) Awareness of conducting assessment
		2) Effective ways of promoting assessment	
		3) Arrange for assessment information system	
Child-adolescent treatment programs after disasters	1. Conducting an intervention	1) Conducting a treatment program	
		2) Essential factors for the treatment program	
	2. Traits of participants	1) Classification of the child-adolescent developmental stage and age	
		2) Division of child-adolescent symptoms	
	3. Treatment program	1) Group therapy	
		2) Time frame of the treatment program	(1) Importance of the time frame of treatment
			(2) Standardization of the Korean version of intervention
		3) Treatment program	(1) TF-CBT
			(2) EMDR
			(3) TRT
			(4) SSET
			(5) C-First Aid
			(6) Play therapy
			(7) Art therapy
			(8) Other interventions
		4) Customized programs for symptom levels	
		5) Family program	(1) Family participation program
			(2) Family camp and crash overnight camp
			(3) Ways of selecting program participants
		6) Standardization of the Korean version of foreign treatment programs	
	4. Facilities in disaster interventions	1) Providing situations for therapeutic intervention	(1) Arranging the place for child-adolescent intervention
			(2) Constructing an environment for intervention
			(3) Providing treatment program information
		2) Opportunities for the treatment program (time, place)	
		3) Keeping materials and artwork in the treatment room	
		4) Recognition of differences and complementary cooperation	(1) Recognition of differences in the related organization
			(2) Complementary cooperation with organization
	5. Treatment professionals	1) Methods for practical use of disaster professionals	
		2) Professionals	(1) Level of professionals
			(2) Qualification of professionals
		3) Arrangement of child-adolescent disaster professionals	
		4) Continuing education system construction for child-adolescent disaster professionals	
	6. Promoting treatment programs	1) Participation in treatment	(1) Continuing participation system for children and adolescents
			(2) Awareness of participation in a treatment program
			(3) Education to continuously participate in treatment regularly
		2) Effective ways of promoting a system	
		3) Creation of protocol information	

Tables 4 and 5 show the evaluation items and intervention items, respectively, for Round 2.

**Table 4:** Contents of post-disaster evaluation in the Round 2 survey.

Variable	Item	CVR	Mean	SD
Concept	Unique model for other psychopathologies	0.875	4.440	0.629
Recovery of Trauma	Return to daily lives	0.500	4.000	0.730
	Attainment of developmental tasks	0.875	4.250	0.775
Screening	Importance of screening	0.875	4.310	0.602
Subject of screening	Self-report	1.000	4.500	0.516
	Family or caregiver report	0.750	4.250	0.856
	Acquaintance or friend report*	0.250	3.560	0.814
	Teacher report	1.000	4.440	0.512
Contents of screening	Checking for coping resources and psychosocial crisis	1.000	4.500	0.516
	Time for 20 minutes	0.125	3.560	0.892
	Interview with family	0.500	3.880	0.957
	Precautions*	0.625	4.130	0.719
	Fill out the developmental progress report	0.000	3.310	0.946
Subject of screening for the high-risk group	Self-report	1.000	4.560	0.512
	Family or caregiver report	1.000	4.690	0.479
	Acquaintance or friend report	0.500	4.000	0.730
	Teacher report	1.000	4.500	0.516
Evaluation of the high-risk group	Required information	1.000	4.560	0.512
	Duration of 60 minutes	0.250	3.810	0.911
	Interview with family	1.000	4.440	0.512
	Precautions*	0.500	4.060	0.772
Scale recommended in screening	Trauma-related scale	0.875	4.690	0.602
	Grief scale	0.625	4.380	0.957
	Depression/anxiety scale	0.875	4.560	0.629
	Suicide scale	0.875	4.560	0.629
	Drug-related scale	0.500	4.190	0.834
	Physical symptom scale	0.750	4.380	0.719
	Social resource scale	0.750	4.380	0.885
	Family function scale	0.500	4.130	0.957
	Adaptation to daily life scale	0.750	4.130	0.957
	Additional required evaluation*	-0.250	3.380	1.088
Things to consider for screening	Fewer than20 questions per scale*	0.500	4.000	1.033
	In the individual evaluation, a total of 40–50 questions*	0.125	3.810	1.109
	In the group evaluation, a total of 80–100 questions*	-0.375	2.810	1.328
Scales and test recommended for the high-risk group	Trauma-related scale	1.000	4.750	0.447
	Grief scale	0.875	4.560	0.629
	Depression/anxiety scale	1.000	4.690	0.479
	Suicide scale	1.000	4.690	0.479
	Drug-related scale	0.625	4.250	0.931
	Physical symptom scale	0.875	4.500	0.632
	Social resource scale	1.000	4.750	0.447
	Family function scale	0.875	4.630	0.619
	Adaptation to daily life scale	0.875	4.630	0.619
	Intelligence test	-0.125	2.940	1.436
	Projection test*	-0.500	2.380	1.455
	Neuropsychological test	-0.500	2.500	1.414
	Additional required evaluation*	-0.375	3.500	1.155
Things to consider in the high-risk group	Less than20 questions per scale*	0.125	3.380	1.455
	In the individual evaluation, a total of 40–50 questions*	0.375	3.880	1.204
	In the group evaluation, total of 80–100 questions*	-0.125	3.250	1.390
Disaster evaluation professionals	Importance of disaster evaluation professionals	0.500	4.060	1.063
	Professional qualifications and levels	0.875	4.310	0.602
	Application plan for disaster assessment professionals	0.625	4.060	1.181
	Inclusion in professional education curriculum	0.875	4.560	0.629
Promoting a plan for evaluation processes	Importance of promoting evaluation*	1.000	4.440	0.512
	Awareness of conducting assessment	1.000	4.560	0.512
	Education in school	1.000	4.500	0.516
	Campaigns on public TV	0.875	4.500	0.632
	Advertisement on the Internet	0.875	4.310	0.602
	Advertisement on SNSs	0.625	4.060	0.680
	Advertisement in education offices	0.625	4.250	0.775
	Advertisement in the community	0.500	4.000	0.894
	Prior education	0.875	4.380	0.619
	Parents’ education	0.750	4.500	0.894
	Teacher education	1.000	4.810	0.403

* Excluded (low CVR) items in Round 3.

**Table 5:** Contents of post-disaster intervention in the Round 2 survey.

Variable	Item	CVR	Mean	SD
Conducting treatment programs	Importance of intervention	0.750	4.440	0.892
	Psychoeducation after disaster	1.000	4.810	0.403
	Guideline for coping with the media	1.000	4.810	0.403
	Normalization/stabilization education	0.875	4.690	0.602
	Practice for physical stabilization	0.875	4.560	0.629
	Classification by acute/maintenance intervention	1.000	4.750	0.447
	Education for families	1.000	4.810	0.403
	Education for teachers	1.000	4.810	0.403
	Handling of guilt	0.750	4.690	0.704
	Dealing with emotion	0.875	4.630	0.619
Time frame	1–4 sessions	0.500	4.060	0.929
	5–8 sessions	0.125	3.880	0.885
	9–12 sessions*	-0.250	3.250	0.856
	Long-term sessions*	-0.500	2.750	1.125
Time of intervention	Immediately after disaster, interventions as quick and as brief as possible	0.875	4.440	1.031
	If there is physical trauma, intervene after pain relief*	0.000	3.310	1.250
	Classification of acute/sub-acute/chronic stage	1.000	4.690	0.479
Termination of session	If both therapist and client agree*	0.250	3.690	1.014
	If the client feels he or she has recovered*	0.125	3.440	1.263
Subject of intervention	Categorization by developmental stage/age	1.000	4.810	0.403
	Division of child-adolescent symptoms	0.750	4.440	0.727
	Combining individual therapy with group therapy	0.250	3.810	1.167
Number of participants in group sessions	2–4*	0.250	3.690	0.946
	5–8*	0.250	3.810	0.750
	9–12*	-0.750	2.750	0.683
	13–16*	-0.875	2.060	1.063
	Whole class*	-0.875	2.000	0.894
Time frame	For toddlers and preschoolers, 20 minutes with parent participation	0.250	3.630	0.885
	In lower grades of elementary school, 30–40 minutes	0.875	4.190	0.544
	In upper grade of elementary school, 40 minutes	0.875	4.190	0.544
	In middle/high school, 45–50 minutes	0.875	4.130	0.500
Treatment program	Importance of intervention guidelines	0.875	4.560	0.629
	PFA	0.875	4.560	0.629
	TRT	0.625	3.880	0.500
	SSET	0.250	3.690	0.602
	TF-CBT	0.375	3.810	0.834
	EMDR	0.125	3.560	0.727
	PE	-0.250	3.250	0.856
	Trauma-focused play therapy*	-0.125	3.250	0.856
	Trauma-focused art therapy*	-0.375	3.060	0.929
	Family program	0.875	4.310	0.602
	Additional programs needed*	-0.625	3.310	0.704
South Korean version of toddler and preschooler therapy	Necessity for standardization in the Korean version	0.750	4.250	0.683
	PFA	0.875	4.250	0.775
	TRT	0.500	3.880	0.957
	SSET*	0.000	3.310	1.302
	TF-CBT*	0.000	3.250	1.390
	EMDR*	-0.250	2.940	1.340
	PE*	-0.125	3.060	1.289
	Trauma-focused play therapy*	0.125	3.630	0.957
	Trauma-focused art therapy*	-0.125	3.130	1.258
	Additional programs needed*	-0.625	3.250	0.775
South Korean version of grade-schooler therapy	Necessity for standardization in the Korean version	0.750	4.380	0.719
	PFA	1.000	4.500	0.516
	TRT	0.500	4.060	0.772
	SSET*	0.125	3.810	0.834
	TF-CBT	0.375	4.000	0.816
	EMDR	0.250	3.690	1.014
	PE*	0.125	3.630	1.025
	Trauma-focused play therapy*	0.000	3.440	1.153
	Trauma-focused art therapy*	-0.250	3.130	1.204
	Additional programs needed*	-0.625	3.250	0.775
South Korean version of middle/high school therapy	Necessity for standardization in the Korean version	0.875	4.440	0.629
	PFA	0.875	4.250	0.577
	TRT	0.625	4.060	0.854
	SSET	0.625	4.060	0.680
	TF-CBT	0.750	4.250	0.683
	EMDR	0.500	3.940	0.854
	PE*	0.125	3.750	0.775
	Trauma-focused play therapy*	-0.125	3.130	1.204
	Trauma-focused art therapy*	0.125	3.310	1.195
	Additional programs needed*	-0.625	3.250	0.775
Facilities in disaster interventions	Arrange the place for child-adolescent intervention	1.000	4.560	0.512
	Providing treatment program information	1.000	4.500	0.516
	Treatment program opportunities	0.625	4.250	0.775
	Acceptance of in-school counseling as a class*	0.375	4.060	1.289
	Arrangement of materials	0.500	3.940	0.998
	Complementary cooperation with organization	1.000	4.560	0.512
Disaster intervention professionals	Importance of disaster intervention professionals	0.750	4.380	0.885
	Professional qualifications and levels	0.875	4.440	0.814
	Need for all mental health workers to conduct treatment	0.750	4.310	0.873
	Completion of disaster care curriculum	0.875	4.560	0.629
	Knowledge of secondary traumatizations	1.000	4.690	0.479
	Education system for disaster intervention professionals	1.000	4.690	0.479
	Continuous supervision	1.000	4.690	0.479
Promoting treatment programs	Continuing the system for child-adolescent participation	0.750	4.380	0.719
	Creation of a system for referrals to therapy*	-0.250	3.250	1.238
	Education of the whole school	1.000	4.630	0.500
	Support for medical expenses from the government	0.875	4.560	0.629
	Decrease in the stigma of psychiatric treatment	0.500	4.190	0.834
	Cooperation with the community	1.000	4.630	0.500

* Excluded (low CVR) items in Round 3.

In the conceptual and semantic domain of trauma in children and adolescents, the CVR was 0.49 or higher, and the content validity was verified for all items. The average value and the CVR were the highest in the ‘self-report’ and ‘teacher-report’ assessments. In contrast, the CVR for ‘the importance of evaluating an acquaintance (or a friend) of victims from the disaster’ was less than 0.49 (Table 4).

The screening questionnaire items ‘necessary to meet a family member at the time of screening’ and ‘caution when interviewing children and adolescents’ were validated. The CVR was the highest for ‘trauma, depression, anxiety, suicide, physical symptoms, social support, adaptation, and mood response should be included in the screening test’. Nevertheless, the CVR was less than 0.49 for ‘20 minutes of screening time is needed’ and ‘children's developmental considerations must be considered’. Therefore, the items with low CVRs were excluded in the third round, and supplementary items were developed (Table 4).

In the high-risk group, the CVR was highest for ‘child, adolescent, family, teacher evaluation’. However, the CVR for the item ‘It takes about one hour to interview the high-risk group’ was less than 0.49. Based on an additional comment from the expert panels, it was decided that the third round should include ‘30 minutes to 1 hour is most appropriate when evaluating a high-risk group’. In addition, many opinions suggested that ‘they should evaluate trauma, depression, anxiety, suicide, and social support’. However, the item ‘intelligence, projection test, and neuropsychological evaluation are necessary’ was excluded from the third round because the CVR was less than 0.49 (Table 4).

In addition, the CVR was lower than 0.49 for ‘the number of program sessions is “5 to 8 sessions”, “9 to 12 sessions”, and “13 sessions or more” is required’ if the intervention program is implemented after a disaster. The CVR was also low for ‘the treatment was terminated if the child had recovered the level of functioning’. These items should be excluded because of CVR validity; however, we revised those items based on additional comments from the experts, and the revised items were used in the third round (Table 5).

The CVR for the ‘need for standardized PFA (psychological first aid) and TRT (teaching recovery techniques)’ for the Korean version for infants and children was higher than 0.49. However, the CVRs for ‘SSET (support for students exposed to trauma), TF-CBT (trauma-focused cognitive behavior therapy), EMDR (eye movement desensitization and processing), PE (prolonged exposure therapy), trauma-focused play therapy and art therapy’ were low. In this case, the opinion of experts on Korean culture was reflected in the third round. However, the need for the Korean version of the PFA, TRT, SSET, TF-CBT, and EMDR was associated with a CVR higher than 0.49 (Table 5).

### Results of third-round Delphi survey

The evaluation items and intervention items for Round 3 are described in detail in Tables 6 and 7, respectively.

**Table 6:** Contents of post-disaster evaluation in the Round 3 survey.

Variable	Item	CVR	Mean	SD
Concept	Unique model for other psychopathologies	0.917	4.375	0.711
Recovery of Trauma	Return to daily lives	1.000	4.750	0.442
	Attainment of developmental tasks in a long-term stage*	0.833	4.167	0.565
	Disappearance of reactions and symptoms of trauma**	0.250	3.708	0.624
	Stabilization of social functioning**	0.583	3.917	0.584
	Stabilization of relationships**	0.583	3.875	0.637
	Stabilization of academic functioning**	0.250	3.625	0.770
Significance	Guarantee of the usefulness of exceptions in screening*	0.833	4.208	0.588
Subject of screening	Self-report	0.917	4.583	0.584
	Family or caregiver report	0.750	4.167	0.637
	Teacher report	0.750	4.167	0.637
Contents of screening	Checking for coping resources and psychosocial crisis	0.917	4.417	0.584
	Duration of 10–15 minutes*	0.833	4.375	0.647
	Explanation of brief care service**	0.917	4.292	0.550
	Interview with family	0.000	3.458	0.833
	Screening at moderate speed**	1.000	4.375	0.495
	Importance of attitude of mind**	0.917	4.417	0.584
	Concern for secondary damage**	1.000	4.708	0.464
	Importance of safety and mutual trust**	1.000	4.833	0.381
	For toddlers and preschoolers, fill in developmental progress*	0.417	3.917	0.717
	Understanding previous traumatic experience**	1.000	4.292	0.464
	Checking for separate experiences of parents**	0.167	3.583	0.881
Scale recommended in screening	Trauma-related scale	1.000	4.708	0.464
	Grief scale	0.833	4.458	0.658
	Depression/anxiety scale	1.000	4.542	0.509
	Suicide scale	0.917	4.542	0.721
	Drug-related scale	0.333	3.917	0.974
	Addiction scale**	0.333	3.875	0.947
	Physical symptom scale	0.917	4.417	0.584
	Sleep-related scale**	1.000	4.500	0.511
	Social resource scale	0.667	3.958	0.806
	Family function scale	0.417	3.792	1.021
	Adaptation to daily life scale	0.500	4.000	0.834
	Existing psychological problem scale**	0.583	3.917	0.929
Things to consider in screening	Minimal screening question**	0.833	4.417	0.654
	Question of the prediction of a high-risk group**	1.000	4.667	0.482
Subject of screening for the high-risk group	Self-report	0.917	4.667	0.565
	Family or caregiver report	0.917	4.500	0.590
	Acquaintance or friend report	0.083	3.625	0.875
	Teacher report	0.750	4.292	0.690
Evaluation of the high-risk group	Environment of safety and stabilization**	1.000	4.750	0.442
	Information on medical history and symptoms*	0.833	4.417	0.654
	Duration of 30–60 minutes*	0.833	4.375	0.647
	Interview with family	0.917	4.417	0.584
	Checking for psychological crisis in the family**	0.833	4.333	0.637
Scales and tests recommended for the high-risk group	Psychiatric interview**	0.917	4.542	0.588
	Trauma-related scale	1.000	4.667	0.482
	Grief scale	0.833	4.417	0.654
	Depression/anxiety scale	1.000	4.500	0.511
	Suicide scale	1.000	4.667	0.482
	Drug-related scale**	0.667	4.250	0.847
	Addiction scale**	0.500	4.042	0.859
	Physical symptom scale	0.917	4.417	0.584
	Sleep-related scale**	1.000	4.583	0.504
	Social resource scale	1.000	4.417	0.504
	Family function scale	0.833	4.250	0.608
	Adaptation to daily life scale	0.833	4.292	0.624
	Assessment of school record**	0.333	3.792	1.103
	Intelligence test	0.417	3.708	1.083
	Existing psychological problem scale**	0.833	4.333	0.637
	Assessment of family’s medical history**	0.417	3.917	0.830
	Neuropsychological test	0.667	4.083	0.881
	Assessment of crisis management ability**	0.667	4.208	0.721
Things to consider in the high-risk group	Importance of personal interviews**	1.000	4.583	0.504
	Evaluation in a safe place**	1.000	4.500	0.511
Disaster evaluation professionals	Importance of disaster evaluation professionals	0.917	4.333	0.565
	Application plan for disaster assessment professionals	0.750	4.417	0.717
	The need for all mental health workers to conduct assessment**	-0.333	3.167	0.963
	Professional qualifications and levels	1.000	4.458	0.509
	Training on crisis management in disasters**	0.917	4.292	0.550
	Upgrading the quality of professionals**	1.000	4.583	0.504
	Importance of having clinical experience**	1.000	4.625	0.495
	Education system construction for child-adolescent disaster professionals**	0.917	4.417	0.584
	Inclusion in professional education curriculum	1.000	4.375	0.495
Promoting a plan for evaluation processes	Ways for system of continuous participation in assessment**	0.833	4.250	0.608
	Arrangement of prior information**	1.000	4.458	0.509
	Effective ways for early advertisements to the nation**	0.667	4.125	0.680
	Awareness of conducting assessment	0.917	4.417	0.584
	Top-down system from education offices**	0.417	3.792	0.833
	Setting up guidelines for ethical behavior**	0.917	4.417	0.584
	Audio-visual education at school*	0.833	4.417	0.565
	Campaigns on public TV	0.583	4.208	0.779
	Advertisement on the Internet	0.583	4.083	0.717
	Advertisement on SNSs	0.417	3.917	0.930
	Advertisement from education offices	0.750	4.250	0.794
	Advertisement in the community	0.250	3.874	0.900
	Prior education	0.667	4.208	0.721
	Parents’ education	0.750	4.292	0.690
	Teacher education	0.750	4.500	0.722

* Modified items in Round 3.

** Newly added items in Round 3.

**Table 7:** Contents of post-disaster intervention in the Round 3 survey.

Variable	Item	CVR	Mean	SD
Conducting treatment programs	Importance of intervention	0.833	4.417	0.776
	Effective ways to use a precautionary approach**	0.833	4.250	0.737
	Psychoeducation after disasters	1.000	4.625	0.495
	Guideline for coping with the media	0.917	4.500	0.590
	Normalization/stabilization education	1.000	4.625	0.495
	Practice for physical stabilization	1.000	4.500	0.511
	Handling of guilt	0.917	4.417	0.584
	Classification by acute/maintenance intervention	0.833	4.458	0.658
	Education for families	1.000	4.583	0.504
	Education for teachers	1.000	4.583	0.504
	Dealing with emotion	1.000	4.542	0.509
Subject of intervention	Categorization by developmental stage/age	1.000	4.667	0.482
	Division of child-adolescent symptoms	1.000	4.375	0.495
	Combining individual therapy with group therapy	0.833	4.125	0.680
	Standard of participation and exceptions**	1.000	4.500	0.511
	Classification of traits in groups**	0.833	4.292	0.751
	In group therapy, interventions should differ, depending on the trauma type**	0.917	4.250	0.532
	Conduct disaster intervention on a large scale**	0.417	3.833	0.868
	In general, psychoeducation and education to the whole class**	0.833	4.375	0.647
Time of intervention	Immediately after a disaster, interventions as quick and brief as possible	0.500	4.042	0.859
	About one week after a disaster, planning psychoeducation**	0.583	4.000	0.885
	Classification of acute/sub-acute/chronic stage	0.833	4.292	0.624
	Immediately after a disaster, stabilization/support-centric acute intervention**	1.000	4.458	0.509
	One month after a disaster, trauma-focused intervention**	0.750	4.125	0.612
	Follow-up for the recovery of daily life functioning**	1.000	4.375	0.495
End of session	Improving post-test scores versus screening**	0.167	3.542	1.062
	After fixed session ended, refer to follow-up**	0.667	4.083	0.654
Time frame	In preventing intervention, 1–4 sessions**	1.000	4.542	0.509
	In therapeutic intervention, 1–4 sessions*	0.167	3.417	1.283
	In therapeutic intervention, use 5–8 sessions flexibly*	0.667	4.208	0.833
	For toddlers and preschoolers, 30 minutes with parent participation*	0.750	4.167	0.761
	In lower grades of elementary school, 30–40 minutes	0.917	4.292	0.550
	In upper grades of elementary school, 40 minutes	0.917	4.292	0.550
	In middle/high school, 45–50 minutes	0.917	4.292	0.550
Treatment program	Importance of intervention guidelines	1.000	4.583	0.504
	PFA	1.000	4.542	0.509
	TRT	0.833	4.250	0.737
	SSET	0.833	4.208	0.721
	TF-CBT	0.833	4.333	0.637
	EMDR	0.750	4.208	0.658
	PE	0.500	3.000	0.834
	Family program	0.667	4.042	0.751
	Grief program**	0.667	4.125	0.680
	Personal psychotherapy/medication**	1.000	4.500	0.511
South Korean version of toddler and preschooler therapy	Necessity for standardization in the Korean version	0.917	4.417	0.584
	Verification of the case applied in Korea**	0.833	4.208	0.588
	PFA	0.917	4.292	0.550
	TRT	0.667	4.000	0.722
South Korean version of grade-schooler therapy	Necessity for standardization in the Korean version	0.917	4.500	0.590
	PFA	0.917	4.417	0.584
	TRT	0.833	4.250	0.608
	TF-CBT	0.833	4.208	0.588
	EMDR	0.417	3.792	0.833
South Korean version of middle/high school therapy	Necessity for standardization in the Korean version	1.000	4.458	0.509
	PFA	0.917	4.375	0.576
	TRT	0.917	4.208	0.509
	SSET	0.917	4.250	0.532
	TF-CBT	0.833	4.208	0.588
	EMDR	0.500	3.875	0.741
Facilities in disaster interventions	Arrange a place for child-adolescent intervention	1.000	4.500	0.511
	Providing treatment program information	0.917	4.500	0.590
	Treatment program opportunities	1.000	4.500	0.511
	Keeping materials and artworks in the treatment room**	0.917	4.542	0.588
	Complementary cooperation with organization	1.000	4.500	0.511
	Arrangement of materials	1.000	4.625	0.495
Disaster intervention professionals	Importance of disaster intervention professionals	1.000	4.500	0.511
	Need for all mental health workers to conduct treatment	0.333	3.792	0.884
	Professional qualifications and levels	1.000	4.500	0.511
	Arrangement of disaster professionals**	1.000	4.500	0.511
	Completion of disaster care curriculum	1.000	4.458	0.509
	Knowledge of secondary traumatizations	1.000	4.667	0.482
	Necessity for peer support groups**	0.917	4.583	0.584
	Participation of professionals such as psychiatrists and psychologists**	1.000	4.625	0.495
	Construction of network for in-depth therapy**	1.000	4.583	0.504
	Development of education system for intervention professionals*	1.000	4.542	0.509
	Continuous supervision	0.917	4.375	0.576
	Plan for group/online supervision*	0.917	4.208	0.658
	Setting up an information network*	1.000	4.417	0.504
Promoting treatment programs	Continuing system for child-adolescent participation	0.917	4.583	0584
	Creation of a system for referrals to therapy	1.000	4.500	0.511
	Education of the whole school	1.000	4.542	0.509
	Support for medical expenses from the government	1.000	4.625	0.495
	Cooperation with the community	1.000	4.625	0.495
	Effective ways to promote the system*	0.833	4.250	0.608
	Education to continuously participate in treatment regularly*	1.000	4.417	0.504
	Decrease in the stigma of psychiatric treatment	0.833	4.208	0.588

* Modified items in Round 3.

** Newly added items in Round 3.

The CVR for Round 3 was 0.38 or higher, and the content validity was verified for nearly all items. The major items with high CVRs are described as follows.

The CVRs were higher than 0.38 for the following items: ‘children and adolescents experiencing trauma should adjust to their current life to recover from trauma’, ‘stabilize their social and interpersonal functions’, and ‘fulfill their developmental tasks in the long term’ (Table 6).

In particular, in the high-risk group, the average value and the content CVR were the highest for the item ‘the child and the family should be evaluated’. The highest CVR was observed for the opinion that a trauma-related scale and scales for depression, anxiety, suicide, sleep, and social resources are needed. The CVR was 0.38 or higher for the items indicating that specialists who perform a psychological assessment in a disaster need ‘crisis management training’ and the ‘ability to cope with various responses of clients’ (Table 6).

In terms of the intervention program, the CVR was the highest for ‘psychological education for the post-traumatic response, normalization, stabilization, physical stability training, family and teacher education, and emotion education should be included.’ With respect to the elements of a therapy program, a high CVR was observed for ‘requiring PFA, TRT, SSET, TF-CBT, EMDR, PE, a family participation program, a mourning-themed program, individual psychotherapy and medication’. Opinions suggesting that ‘individual psychotherapy and medication are needed’ were most frequently observed. In addition, some comments indicated that ‘child-parent psychotherapy might be more appropriate than PFA and TRT for toddlers and preschoolers’ (Table 7).

With respect to the termination of therapy, CVRs higher than 0.38 were observed for the following items: ‘the intervention should be terminated after the prescribed therapy sessions’ and ‘referrals should be determined thereafter’ (Table 7).

A high CVR was found for the item regarding the intervention development strategy: 'establish a therapeutic linkage system based on national support, educate and inform the whole school, support medical expenses (such as with government subsidies), connect with the community, consider the persistence of treatment cases, and reduce the stigma of psychiatry’ (Table 7).

## Discussion

In South Korea, the dispute over how to evaluate and intervene in the aftermath of the Sewol Ferry Disaster required a consensus regarding the need for disaster planning.[24] The Delphi process was a suitable method for surveying experts on this topic.[25] Using this method, we propose a multidisciplinary recommendation for treating children exposed to disasters. The results of qualitative and quantitative analyses conducted through the Delphi panel survey demonstrate that psychosocial assessment and intervention are essential to early mental health services following a disaster. We discuss suggestions based on the consensus of the experts involved in the study.

We found that in the event of a disaster, intervention factors such as ‘appropriate time for assessment after the disaster’, ‘prerequisites for screening and in-depth intervention’, ‘classifying the degree of psychosocial symptoms’, and ‘social and mental health services’ are very important. Recovery from psychological trauma after a disaster means mental stability as well as the recovery of physical health. Screening tests are recommended for all children exposed to disasters, particularly during acute periods of disaster. After the completion of screening tests, assessment should include in-depth interviews and interventions for the high-risk group. First, however, we must distinguish between brief screening and in-depth evaluation. As in our study, many previous studies have suggested mental health assessments and interventions for children.[26] These findings are consistent with research findings indicating that screening is appropriate when large numbers of children are exposed to an event or when the level of exposure among a population is unknown.[27]

The actual screening assessment performed after a disaster requires the consideration of each stage of the disaster and should consist of appropriate questions.[28] In the disaster context, screening tools should reflect the needs of children with mental health problems, including consideration of children’s exposure, experience, and subjective reactions to traumatic events and conditions.[29, 30]

Evaluation of children, families, and teachers during the acute phase of a disaster is important. Above all, consensus among experts on the selection of children exposed to a disaster is required. Families and teachers should be evaluated together. The use of multiple informants, such as parents, teachers, and other professionals, as collateral sources of information enables the most comprehensive appraisal of children’s reactions and functioning.[28] These results are consistent with the opinion that it is important for parents and/or caregivers to participate together in a child's treatment session to recover from PTSD symptoms.[26] When interviewing a family member, we must check for signs of psychological crisis among family members. This finding is consistent with studies of the family environment, social support, and supportive quality.[31] However, it is not necessary to evaluate acquaintances or friends. Furthermore, assessments of grief, depression, anxiety, and suicide risk, as well as trauma-related scales, need to measure PTSD and other psychosocial symptoms. This finding is largely consistent with a previous report that disaster exposure is correlated with PTSD, depression, anxiety, functional impairment, and behavioral problems.[32] In addition, trauma assessment of children and adolescents should consider their developmental stage. When treating a child who has experienced trauma, the clinician must understand the child's existing psychopathological symptoms and provide appropriate interventions, such as trauma-focused therapy.[26] Our results suggest the need to develop a crisis intervention model for children and adolescents.[33]

Psychosocial assessments should be conducted in a safe environment and at appropriate durations of 30–60 minutes. Approximately 30 to 60 minutes is needed for screening a high-risk group.

Psychoeducation is also beneficial to children. A post-disaster intervention program should include the following: psychoeducation, guidelines for coping with the media, normalization, stabilization, techniques for handling survivor’s guilt and emotion-focused coping strategies. Appropriate access phases can be classified as hyper-acute, acute, sub-acute or chronic stages. Stabilization and psychological support should be provided immediately after a disaster along with intervention to help children adapt to everyday life. This finding is consistent with a report that most interventions are multimodal, incorporating common elements to educate children, normalize their reactions, process their emotions and manage stress, enhance coping and provide social support.[27] In addition, the development stage, age, trauma symptoms, and traits of a group should be considered. The number of children participating in a group may vary depending on the type of disaster. In general, psychoeducation can be provided in the class setting at school. For prevention education, holding one to four sessions is recommended, whereas for therapeutic intervention, five to eight sessions are appropriate. If the child is exposed to a national large-scale disaster, intervention to address brief trauma may not be sufficient. Therefore, professional intervention should be provided, particularly for children with symptoms of PTSD.[26]

For a preschooler, the appropriate duration of an intervention is 30 minutes with caregiver participation. A proper duration of 30 to 40 minutes is suitable for elementary school students in lower grades. An intermediate duration of 40 minutes is suitable for elementary school students in higher grades. For middle and high school students, intervention programs could last 45 to 50 minutes. The optimal intervention components may not be the same for all children or all situations, which should be examined in future work.[34]

We recommend the following available intervention programs: PFA[35], TRT[36], SSET[37], and TF-CBT[38]. In South Korea, the South Korean versions of PFA, TRT, and TF-CBT should be standardized for children and adolescents. However, the study findings provided no suggestion related to narrative therapy. Furthermore, an intervention for toddlers and preschoolers should be considered. Multiple evidence-based programs should be considered as well, and an intervention protocol that includes a standardized South Korean version can then be implemented. These results provide a framework for further research. Accordingly, the CIDER (Children In Disaster: Evaluation & Recovery) protocol developed by the authors of this study will be made available. Additionally, we must include not only child-focused therapy but also long-term mental health services. These findings are partially consistent with a prior study.[39]

The professionals providing disaster interventions vary with respect to factors such as availability, training, and experience, and the goals and complexity of the intervention differ as well.[27] Nevertheless, affected communities do not have enough therapists trained in evidence-based treatments to be able to provide every child with individual therapy.[39] It is not necessary for all mental health workers to conduct evaluations and interventions after a disaster. Therefore, disaster experts with experience working in a clinical environment should be called upon; a training and education system for professionals is needed. Such professionals may need additional support and guidance to address their own emotional responses.[27] This support can be incorporated into supervision as well as peer support groups. Additionally, the present study shows that good relationships should be cultivated within professional networks of information related to in-depth therapy.

Above all, interventions delivered in groups are particularly well suited for school settings.[27] Schools are among the most important links in the chain of public health education for children and adolescents.[40] School-based interventions should be developed, regular training in disaster safety measures for school personnel should be mandated, and training programs for children should be established. Moreover, teachers should receive advice on coping with emergencies in either their basic teacher training or in-service training. In summary, schools should identify school crisis emergencies and clearly delineate the roles of children and teachers in coping with disaster. Based on the abovementioned considerations, psychiatric and psychological support should be accessible. Additionally, guiding children to use positive coping strategies and encouraging a warm community atmosphere are recommended.[32] Consequently, our confidence in reaching consensus means that we now have a comprehensive framework of competency statements that describe what psychiatric professionals working in the aftermath of a disaster must do. As the National Child Traumatic Stress Network has coordinated collaboration among 10 research development and evaluation sites and 26 community mental health centers across the United States, it is also essential to establish sensible governance between central and local governments, between administrative institutions and institutions that provide services, and between public and civic organizations.[41]

This study proposed effective mental health intervention measures and described the implications for developing a post-disaster evaluation treatment protocol. The main strengths of our study include its responses from a panel of defined experts, good response rates and framework of competencies that describe attributes of professionals working within the disaster field. However, some limitations also need to be recognized.

First, the study findings suggest that children in South Korean cultures require disaster-related psychosocial evaluation and interventions, but modifications may be needed to address other cultural issues.

Second, our expert panel was determined by our approach to sampling. E-mails may not have been distributed by some of the professional groups we contacted, and other experts not publishing their work may have been missed. The rich qualitative and quantitative data obtained from this study are very useful for understanding why certain topics are research priorities.[21]

Third, the experts who conducted psychological intervention at Danwon High School after the Sewol Ferry Disaster in South Korea were all psychiatrists, except for two psychologists.[42] The primary aim was to gather psychiatrists’ opinions and experience from the disaster environment. In Round 1, we had limitations in distinguishing between the related areas of expertise in disaster and trauma for the psychological specialists, and these limitations might be reflected in the medical opinions of the panel.

In conclusion, we suggest the need for informed evidence-based assessments, interventions, and treatments for children and adolescents who experience disasters. This survey presents important opinions from trauma care experts and should be utilized by psychiatrists to develop a meaningful protocol for PTSD assessment and treatment. Hence, the results can be applied to existing and future disaster management.

## Supporting information

S1 Appendix The specific 20 questions in Round 1. (DOCX)
