# Supplementary material for: Development of post-disaster psychosocial evaluation and intervention for children: Results of a South Korean delphi panel survey
Source: PLoS One. 2018 Mar 29;13(3):e0195235. doi: 10.1371/journal.pone.0195235 (PMC5875888; doi:10.1371/journal.pone.0195235)
Supplement: S1 Appendix — (DOCX) [file pone.0195235.s001.docx]

Appendix 1. The specific 20 questions in Round 1

| **No.** | **Contents** |
| --- | --- |
|  | **I. Currently used child-adolescent assessment and treatment protocols in disasters** |
| 1 | What is the effective treatment programs that have been going on in the clinic or proven in previous disasters, and what do you think of the reasons? |
| 2 | What difficulties are you having to carry out when implementing assessment protocols and treatment programs in disasters? |
| 3 | What do you think is needed to promote previous treatment programs for children and adolescents? |
|  | **II. Direction of child-adolescent assessment protocols after disaster** |
| 4 | What do you think is the most important part of the need for psychological assessment and intervention for children and adolescents after a disaster? |
| 5 | How do you think the psychological assessment procedure is appropriate? |
| 6 | When conducting the psychological assessments, what do you think should be focused on in the environment? |
| 7 | When using brief scales, what do you think should be the most important consideration? |
| 8 | What do you think should be the focus of the essential factors when selecting assessment scales? |
|  | **III. Direction of child-adolescent treatment programs after disasters** |
| 9 | What do you think is necessary for the intervention program for children and adolescents after a disaster? What's the reason? |
| 10 | When do you think it is appropriate to start treatment program intervention? What do you think is appropriate on the basis of the judgment of intervention? |
| 11 | When do you think it is appropriate to terminate the treatment program? Based on which criteria do you think is appropriate for judging the termination of treatment? |
| 12 | What kind of programs do you think can be used as a treatment program for children and adolescents? |
| 13 | How do you think it should operate when the treatment is in progress? |
| 14 | Do you think Korean standardization is needed among foreign treatment programs? What's the reason? |
|  | **IV. Things to consider in disaster interventions** |
| 15 | What if you experienced environmental difficulties when you were conducting a psychological assessment or treatment program? |
| 16 | What level of qualification do you think is desirable for the level and qualities of the treatment professionals? |
| 17 | How do you think disaster professionals’ education system for children and adolescents is currently being implemented? |
| 18 | What do you think the ways in which children and adolescents with disaster trauma can continue to participate in treatment programs? |
| 19 | What kind of access do you think will help to effective ways of promoting treatment programs? |
| 20 | Finally, what would you like to suggest or advise on the development of assessment and intervention protocol for children and adolescents? |
